# When do people prefer dominant over prestigious political leaders?

**DOI:** 10.1017/ehs.2021.12

**Published:** 2021-03-01

**Authors:** Ángel V. Jiménez, Adam Flitton, Alex Mesoudi

**Affiliations:** 1 Human Behaviour and Cultural Evolution Group, Department of Biosciences, University of Exeter, Exeter, UK; 2 Centre for Culture and Evolution, Department of Psychology, Brunel University London, Uxbridge UB8 3PHUK

**Keywords:** prestige, dominance, social hierarchy, political leadership, populism, right-wing populism, Donald Trump, Hillary Clinton

## Abstract

Previous research has sought to explain the rise of right-wing populist leaders in terms of the evolutionary framework of dominance and prestige. In this framework, dominance is defined as high social rank acquired via coercion and fear, and prestige is defined as high social rank acquired via competence and admiration. Previous studies have shown that right-wing populist leaders are rated as more dominant than non-populist leaders, and right-wing populist/dominant leaders are favoured in times of economic uncertainty and intergroup conflict. In this paper, we explore and critique this application of dominance–prestige to politics. First, we argue that the dominance–prestige framework, originally developed to explain inter-personal relationships within small-scale societies characterised by face-to-face interaction, does not straightforwardly extend to large-scale democratic societies which have frequent anonymous interaction and complex ingroup–outgroup dynamics. Second, we show that economic uncertainty and intergroup conflict predict a preference not only for dominant leaders, but also for prestigious leaders. Third, we show that perceptions of leaders as dominant or prestigious are not fixed, and depend on the political ideology of the perceiver: people view leaders who share their ideology as prestigious, and those who oppose their ideology as dominant, whether that ideology is liberal or conservative. Fourth, we show that political ideology is a stronger predictor than economic uncertainty of preference for Donald Trump vs Hillary Clinton in the 2016 US Presidential Election, contradicting previous findings that link Trump's success to economic uncertainty. We conclude by suggesting that, if economic uncertainty does not directly affect preferences for right-wing populist leaders, other features of their discourse such as higher emotionality might explain their success.

## Graphical abstract

**Figure:**
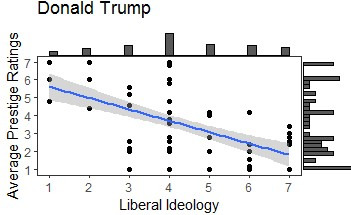

**Social media summary:** New study shows the limitations of current use of dominance–prestige to explain the rise of right-wing populist leaders.

## 1. Introduction

The last two decades have seen the rise of several right-wing populist leaders in democratic countries (Tartar, 2017) such as Donald Trump, Marine Le Pen, Viktor Órban, Matteo Salvini, Nigel Farage, Narendra Modi, Jair Bolsonaro and Geert Wilders. According to Mudde and Kaltwasser (2017), populists share a political discourse that divides society into two antagonistic groups: ‘pure’ people vs the ‘corrupt’ political, economic, cultural and media elite or establishment. They often criticise representative liberal democracy and argue that politics should be a direct expression of the will of the people (e.g. via referenda). Populism is not a complete ideology in itself, unlike socialism or fascism, but it attaches to other ideologies on the right or the left (Mudde & Kaltwasser, 2017).

The *dual evolutionary model of social hierarchy* (Cheng & Tracy, 2014; Cheng et al., 2013; Henrich & Gil-White, 2001; Jiménez & Mesoudi, 2019b; Redhead et al., 2018b) has recently been used to understand the rise of such right-wing populist leaders (Kakkar & Sivanathan, 2017; McAdams, 2017). This model distinguishes between dominance and prestige as two independent strategies that people use to acquire high social rank and influence (Henrich & Gil-White, 2001). The *dominance strategy* entails the use of force and coercion against others. Its success depends on the capacity to defeat and/or induce *fear* in other individuals (Redhead et al., 2018c). Consequently, people tend to dislike dominant individuals (Brand & Mesoudi, 2019; Cheng et al., 2013) and avoid proximity to them (Henrich & Gil-White, 2001). In contrast, the *prestige strategy* entails the display of competence within valued domains and/or pro-ingroup behaviours. Its success depends on the capacity to induce *admiration* and voluntary deference in others because prestigious individuals are perceived as having instrumental value to accomplish one's own goals (Leary et al., 2014), such as socially learning valuable knowledge/skills (Henrich & Gil-White, 2001; Jiménez & Mesoudi, 2019a) or being provided with tangible private (Pinker, 1998, p. 499) and public goods (Price & Van Vugt, 2014). This explains why people generally like and prefer prestigious individuals as both leaders and social companions (Cheng et al., 2013; Kruger & Fitzgerald, 2011; Laustsen & Bor, 2017; Petersen & Laustsen, 2019).

Kakkar and Sivanathan (2017) equated right-wing populist leaders with dominant political leaders. They argue that certain environmental contexts, in particular economic uncertainty, might reverse the preference for prestigious over dominant individuals, at least when choosing between different political leaders. According to these authors, economic uncertainty induces in people a sense of lack of personal control, prompting them to find ways to compensate for that deficit. One of these compensatory strategies is to support dominant political leaders (Hogg & Adelman, 2013), who are perceived as able to defend the interests of the ingroup even at the expense of the well-being of outgroups (Halevy et al., 2012).

Consequently, Kakkar and Sivanathan (2017) attribute the rise of right-wing populist leaders to the rise in economic uncertainty after the 2008 global financial crisis (see also Funke et al., 2016; Funke & Trebesch, 2018). First, Kakkar and Sivanathan found that, during the 2016 US Presidential Campaign, participants rated the right-wing populist leader Donald Trump significantly higher in dominance and lower in prestige than the opposing candidate, Hillary Clinton. Supporting the link to economic uncertainty, Kakkar and Sivanathan further showed that (a) individual voters’ preference for Donald Trump before the election was significantly predicted by an aggregate zip-code-based measure of economic uncertainty composed of housing vacancy rate, poverty rate and unemployment rate and (b) at a country level, using data from the World Values Survey (WVS) from 2004 to 2016 (Inglehart et al., 2018) with 138,323 respondents from 69 countries, preference for dominant leaders in general (a preference for ‘a strong leader who does not have to bother with parliament and elections’) was significantly predicted by the change of unemployment from one year to the next within that country according to the World Bank.

Theories and findings such as those of Kakkar and Sivanathan (2017) are valuable in integrating across disciplines (psychology, economics, politics and anthropology) and providing novel explanations for socially and politically important trends. However, precisely because of this importance, such claims and evidence should be carefully evaluated and scrutinised. In this article, we critically evaluate the above theory and evidence linking right-wing populist leaders to dominance via economic uncertainty. We first discuss the limitations of the current applications of the dual evolutionary model of social hierarchy to modern politics and the rise of right-wing populism, arguing for a greater role of political ideology. Second, we formulate a number of hypotheses derived from the limitations we identify. Third, we test these hypotheses using data from the WVS as well as the data collected by Kakkar and Sivanathan (2017) to analyse the perceptions of and preferences for Donald Trump and Hillary Clinton. Finally, we discuss our results in the context of the broader literature on prestige–dominance, political psychology and populism.

### 1.1. The dual evolutionary model of social hierarchy as applied to politics

Although originally conceived as an explanation for social rank hierarchies within small, face-to-face groups, the prestige–dominance distinction has also been applied to the political arena in large-scale societies, as described above for the 2016 US Presidential Election (Kakkar & Sivanathan, 2017; McAdams, 2017; Witkower, 2017). In these applications, Donald Trump is depicted as dominant because of his more aggressive vocabulary, threats against political rivals (e.g. ‘lock her up’, referring to his election rival Hilary Clinton), non-verbal displays of dominance such as occupying more space and extending his arms (Witkower, 2017), and the display of emotional and personality traits associated with dominance (Cheng et al., 2010) such as high neuroticism, low agreeableness and hubristic pride (McAdams, 2017; Nai et al., 2019). Conversely, Hillary Clinton is depicted as prestigious because of her greater political experience and expertise (e.g. having previously been Secretary of State) as well as her more frequent demonstrations of knowledge and non-verbal displays associated with the prestige strategy (e.g. smiling) during the Presidential debates (Witkower, 2017).

However, the application of the dual evolutionary model of social hierarchy to modern politics and the rise of right-wing populist leaders is not without difficulties. The model was initially developed to explain the acquisition of high social rank and social influence within small groups (e.g. hunter–gatherer bands or sports teams) in which members interact face-to-face and personally know each other. In contrast, the acquisition of political power in modern nation-states occurs within large populations (e.g. the population of the USA exceeds 325 million people) in which members only interact with and know a small proportion of other members. Although many political decisions are taken within small groups (e.g. members of a government), the acquisition and maintenance of political power and political influence within the modern political sphere are more complex than within the small groups to which the model was originally applied, for a number of reasons.

First, the key distinction between the dominance and prestige strategies in the dual evolutionary model is that the social influence attained through dominance is imposed upon others, while the social influence attained through prestige is voluntarily given by others (de Waal-Andrews et al., 2015; Henrich & Gil-White, 2001; Van Vugt & Smith, 2019). However, democratic procedures like the presidential elections that led to the victory of Donald Trump are especially tuned to the prestige strategy given that political power is voluntarily given to the party or coalition of parties that has the greatest freely conferred support within a society. Given the fact that Donald Trump attained the US Presidency through democratic elections, not through a coup d’état, labelling his strategy to power ‘dominance’ is questionable. Furthermore, his supporters respected and admired him for his success as a businessman and a negotiator before the election and found him humorous, likable, and trustworthy. These characteristics clearly describe Donald Trump as a prestigious leader, not a dominant one, among his constituency.

Second, the dominance and prestige strategies in the original model were assumed to be displayed towards other members of the ingroup, such as other members of a small-scale society like the !Kung or the Yanomamo (Henrich & Gil-White, 2001), sport teams (Cheng et al., 2010) and other community groups (Brand & Mesoudi, 2019). In modern politics, however, the use of both strategies is often directed towards outgroups, such as when political leaders of one country pursue a military attack or trade war against another country (potentially a dominance strategy) or when leaders make reforms in their own country to gain admiration and be emulated by the international political community (potentially a prestige strategy). However, it is not clear that the concepts of prestige and dominance straightforwardly translate to this intergroup context. For example, a dominant action by a political leader directed towards an outgroup (e.g. bombing another country) might lead to the acquisition of prestige among ingroup members (e.g. by the demonstration of commitment to protect the ingroup from external threats). When facing intergroup conflict, research has found that people increase their preferences for male leaders who have traits associated with the use of the dominance strategy such as facial masculinity, muscle strength, and height (Laustsen & Petersen, 2017; Little et al., 2007; Petersen & Laustsen, 2019), features that people presumably perceive as facilitating the use of aggression against outgroups during intergroup conflict (Laustsen & Petersen, 2017). For instance, Little et al. (2007) found that people prefer hypothetical election candidates with more physically dominant faces during war time and less physically dominant faces during peace time. However, this and other studies have not tested whether intergroup conflict simultaneously increases people's preferences for prestigious political leaders. It is plausible that prestigious leaders are desirable in intergroup conflict because they are more knowledgeable about international relations, or more skilled negotiators and therefore achieve the best deals possible for their ingroup preventing much of the damage of a long-lasting intergroup conflict. Importantly, people might prefer leaders with features usually associated with the dominance strategy, such as physical formidability in men, owing to the perceived competence of these men in between-group representation and ingroup leadership, which in fact better describes prestigious leaders, as shown by Lukasziewski et al. (2016).

Furthermore, although members of one's country might often be perceived as the ‘ingroup’, the existence of different ideologies within a country often leads to conflicts, and groups with opposing ideologies within the same country become perceived as outgroups. This has consequences for the perceptions of dominance and prestige of political leaders and political decisions, as the same decisions might be seen differently by people within the same country but belonging to different ideological groups. For instance, the Catalonian Independence Referendum (1 October 2017) carried out by the pro-independence Catalonia regional government despite being declared illegal by the Constitutional Court of Spain was considered a coup d’état (i.e. an act of dominance) by people who defend the territorial integrity of Spain (e.g. Wintour, 2017), but a democratic process capturing the will of the people (i.e. a prestigious act) by defenders of the independence of Catalonia (e.g. Asamblea Nacional Catalana, 2018). Conversely, the application of article 155 of the Spanish Constitution by the Spanish Government, which resulted in the control of the Catalonian regional power by the Spanish government, was considered a coup d’état (i.e. an act of dominance) by people in favour of the independence of Catalonia, but a reestablishment of democracy (i.e. a prestigious act) by defenders of the territorial integrity of Spain (e.g. Burgen, 2017).

The same applies to the perceptions of liberal/left-wing (e.g. Hillary Clinton) and conservative/right-wing (e.g. Donald Trump) political leaders as either dominant or prestigious. Conservatives might feel that liberal politicians are using political power to impose their views on society by pushing the direction of cultural change towards liberal values. Similarly, liberals might feel that conservative politicians are using political power to impose their views on society by pushing the direction of cultural change towards conservative values. As both conservatives and liberals see political leaders of the opposing ideology as a threat to their personal values, or seeking to coerce them into following alternative values, they might perceive them as dominant. In contrast, political leaders of their own ideology are seen as defending and trying to implement the values that those people view as correct and, therefore, people might perceive these politicians as competent and confer prestige on them. These perceptions of political leaders of one's own ideology as prestigious and political leaders of the opposing ideology as dominant might be exacerbated by the increasing political polarisation experienced in the last two decades, at least in the US (Lukianoff & Haidt, 2018, pp. 128–132).

Third, it is not clear why economic uncertainty would reverse the general tendency of preferring prestigious leaders over dominant leaders as Kakkar and Sivanathan (2017) propose. Although dominant leaders might benefit their ingroup by competing more aggressively over limited resources against outgroups, prestigious individuals, who are assumed to be more competent, might take the right decisions to bring the country out of an economic recession. Therefore, supporting a prestigious leader might also be a way to compensate for the lack of control experienced when facing economic uncertainty.

Furthermore, it is unclear why economic uncertainty would be a better predictor of preference for dominant/right-wing populist leaders than political ideology, as has been claimed (Kakkar & Sivanathan, 2017). Ideology is an alternative explanation for the rise of right-wing populist leaders. This ideological explanation posits that the political discourse and proposed policies of right-wing populist leaders are more attractive for a part of the electorate than the political discourse and policies of well-established political leaders. This explanation is often described as a *cultural or political backlash* against post-materialist political movements such as feminism and environmentalism (Inglehart & Norris, 2016, 2017) and political correctness (Campbell & Manning, 2018, pp. 151–161). Inglehart and Norris (2017) found support for the ideological explanation in a study in which they tested whether ideology or economic uncertainty better accounts for the recent rise of right-wing populism. They used data from the European Social Survey (2002–2014) to predict voting preferences for a right-wing populist party from several control (e.g. age, gender, education), economic (e.g. unemployment, subjective economic uncertainty, living on social benefits) and ideological (e.g. anti-immigration, right-wing self-identification, mistrust in global governance) variables. They found mixed support for the economic uncertainty explanation. For example, right-wing populists were supported more by unemployed people but less by people receiving social welfare. In contrast, all of the ideological predictors (anti-immigration attitudes, mistrust of global and national governance, authoritarian values and right-wing ideology) positively predicted support for populist leaders, giving clear support for the ideological explanation. These results highlight the importance of political values in predicting support for right-wing populist leaders.

Consequently, we suggest that political ideology actually plays a greater role than economic uncertainty in explaining the rise in popularity of right-wing populist leaders. Nevertheless, political ideology and economic uncertainty might interact. Ideology might predict who decides to vote for a right-wing populist leader, but economic uncertainty might have created a window of opportunity for right-wing populists, with their discourse becoming attractive to a greater number of people after the financial crisis.

In conclusion, there exist certain parallels between right-wing populist leaders such as Donald Trump and the dominance strategy (e.g. use of aggressive vocabulary against political rivals). However, there are limitations to this parallel. Donald Trump and other right-wing populist leaders often use this strategy against outgroups or political rivals, as do political leaders of other ideologies. This differs from the dual evolutionary model of social hierarchy in which the dominance strategy is directed towards ingroup members. The use of dominance against outgroups might serve to gain prestige within the ingroup, blurring the original dominance–prestige distinction. Consequently, failing to take the ingroup/outgroup distinction into account might lead to incorrect inferences such as concluding that people prefer dominant political leaders under certain contexts (e.g. economic uncertainty) without considering how political ideologies influence perceptions of dominance and prestige of political leaders (e.g. Kakkar & Sivanathan, 2017) or how dominance strategies against outgroups might confer prestige within ingroups (Halevy et al., 2012).

### 1.2. Hypotheses

Based on the discussion above, we formulated a number of hypotheses (Table 1) that specify the conditions under which dominant and prestigious leaders should be preferred. Hypotheses H1–H3 predict preferences for dominant (H1a, H2a and H3a) and prestigious (H1a, H2b and H3a) political leaders as a result of experiencing economic uncertainty (H1), perceived lack of control (H2) and intergroup conflict (H3). These hypotheses are not mutually exclusive. Dominance and prestige have been conceptualised as two independent strategies to acquire high social rank and social influence through different means (Cheng & Tracy, 2014; Cheng et al., 2013), although the extent this is true in all contexts remains an open question (Jiménez & Mesoudi, 2019b). Consequently, economic uncertainty, perceived lack of control and intergroup conflict might predict preference for both dominant and prestigious political leaders. Hypotheses H4–H6 derive from our argument that political ideologies influence perceptions of political leaders as either dominant or prestigious and the greater importance that we ascribe to political ideology over economic uncertainty in predicting voting preferences. These hypotheses are tested in Studies 1 and 2 described below, which extend and improve on the methods and analyses of previous studies (e.g. Kakkar & Sivanathan 2017) that partially address only some of the hypotheses.

**Table 1.** Hypotheses. H1–H3 refer to the relationship between economic uncertainty, perceived lack of control and intergroup conflict, and preferences for both dominant and prestigious political leaders (Study 1). H4 and H5 refer to how people's political ideology is related to the perceptions of political leaders as dominant or prestigious (Study 2). Because the scale used to measure political ideology ranges from conservative (1) to liberal (7), these hypotheses could also be framed as ‘conservative ideology is negatively related to perceptions of conservative political leaders as dominant and positively related to perceptions of liberal leaders as dominant’ (H4) and ‘conservative ideology is negatively related to perceptions of liberal political leaders as prestigious and positively related to perceptions of conservative leaders as prestigious’ (H5), respectively. H6 is related to whether political ideology is a better predictor of voting decisions than economic uncertainty or not

Economic uncertainty	H1a: Economic uncertainty positively predicts preferences for a dominant leader
	H1b: Economic uncertainty positively predicts preferences for a prestigious leader
Perceived lack of control	H2a: Perceived lack of control positively predicts preferences for a dominant leader
	H2b: Perceived lack of control positively predicts preferences for a prestigious leader
Intergroup conflict	H3a: Intergroup conflict positively predicts preferences for a dominant leader
	H3b: Intergroup conflict positively predicts preferences for a prestigious leader
Perceptions of conservative political leaders	H4: Liberal ideology is positively related to perceptions of conservative political leaders as dominant and negatively related to perceptions of liberal leaders as dominant
Perceptions of liberal political leaders	H5: Liberal ideology is positively related to perceptions of liberal political leaders as prestigious and negatively related to perceptions of conservative leaders as prestigious
Voting decisions	H6: People's political ideology is a stronger predictor of people's voting decisions than economic uncertainty

## 2. Study 1

### 2.1. Introduction

The aim of Study 1 is to test whether economic uncertainty (H1a and H1b), perceived general lack of control (H2a and H2b) and intergroup conflict (H3a and H3b) positively predict preferences for dominant and prestigious leaders respectively. We use data from the longitudinal WVS (Inglehart et al., 2018) for the period 2010–2016. H1a and H2a have been previously tested with this dataset by Kakkar and Sivanathan (2017), but with a longer timeframe, 2004–2016. We are using the data from 2010–2016 because only this period contains all of the variables of interest. Hypotheses H1b, H2b, H3a and H3b (related to prestige and intergroup conflict) have not previously been addressed.

Kakkar and Sivanathan (2017) used a four-point Likert item that asked respondents their opinion of ‘having a strong leader who does not bother with parliament or elections’ (1 = very good, 4 = very bad, reverse coded) as a measure of *preference for a dominant leader*. For *perceived general lack of control*, they used a 10-point Likert item, which asked respondents to indicate how much freedom of choice and control they have over the way their lives turn out (1 = no choice at all, 10 = a great deal of choice, reverse coded). They also used five control variables from the WVS: subjective social class, gender, age, political ideology and income category. However, they did not use any of the economic uncertainty variables included in the WVS such as items about how worried participants were about the possibility of losing or not finding a job, or how often in the last 12 months the participants or their family had gone without enough food to eat. Instead, as their measure of *economic uncertainty*, they used the change in unemployment in a country from one year to the next, which was extracted from the Word Development Indicators database from the World Bank. They found support for our hypotheses H1a and H2a (Table 1): both economic uncertainty and perceived general lack of control positively predicted preference for a dominant leader. However, the adjusted *R*^2^ was very low (*Adj R*^2^ = 0.002) and there was no difference in the adjusted *R*^2^ between the model including only the control variables and the models that also included economic uncertainty alone or together with perceived general lack of control. This might be the consequence of using the same value of economic uncertainty (i.e. change in unemployment) for all of the respondents from the same country within a year, which totally eliminates the variation in economic uncertainty between individuals in the same country. To improve on their analysis, we use individual-level variables extracted from the WVS to measure economic uncertainty. Moreover, we adopt a model comparison approach to compare the strength of economic uncertainty, perceived general lack of control and intergroup conflict in predicting preferences for both dominant and prestigious leaders.

### 2.2. Methods

We used the same item as Kakkar and Sivanathan (2017) to measure the outcome variable *preference for a dominant leader*. Our second outcome variable, *preference for a prestigious leader*, was measured with a four-point Likert item (1 = very good, 4 = very bad) in which respondents gave their opinion about the way of governing: ‘having experts, not government, make decisions according to what they think is best for the country’ (1 = very good, 4 = very bad, reverse coded). We chose this item because it is consistent with Henrich and Gil-White's (2001) prestige definition, which centres on knowledge and skill as key aspects of leadership.

For the predictor variable *economic uncertainty* we used five items. Two of those items asked respondents to indicate how worried they were about the possibility of losing or not finding a job (EcUnJOB) and about not being able to give their children a good education (EcUnEDUCATION) (1 = very much, 4 = not at all, reverse coded). The remaining three items asked respondents to indicate how often in the last 12 months they or their family had gone without enough food to eat (EcUnFOOD), without medicine or medical treatments they needed (EcUnMEDICINE) and without a cash income (EcUnCASH) (1 = often, 4 = never, reverse coded). *Intergroup conflict* was measured with three 4-point Likert items indicating how often respondents are worried about a war involving their country (InConINTWAR), a civil war (InConCIVILWAR) or a terrorist attack (InConTERRORISM) (1 = very much, 4 = not at all, reverse coded). Each item for both the economic uncertainty variable and the intergroup conflict variable was used as a separate predictor to preserve its meaning. This also entailed treating each Likert item as ordinal rather than averaging it and treating it as continuous. *Perceived lack of control* was measured with the same item as Kakkar and Sivanathan (see Section 2.1). As *control variables* we used the same variables as Kakkar and Sivanathan: age, gender, income category (10-point Likert scale from lowest group to highest group within respondents’ country), subjective social class (five-point Likert scale, 1 = upper class, 5 = lower class, reverse coded) and political ideology (10-point Likert item from left to right).

After excluding respondents who did not provide information from one or more of these variables, the dataset contained 52,325 respondents (26,209 females, 26,116 males) aged 16–99 (mean, *M* = 41.27, standard deviation = 15.95) from 54 different countries.

Because both outcome variables (preference for a dominant leader and preference for a prestigious leader) are ordered categorical variables and respondents lived in different countries, we used ordinal mixed effects logistic regression models to analyse the data (Bürkner & Vuorre, 2019) with intercepts varying by country of respondents. As the predictor variables of interest (i.e. the items used as proxies for economic uncertainty, lack of control and intergroup conflict) were ordered categorical variables, we modelled their relationship with the outcome variable as a monotonic effect (Bürkner & Charpentier, 2018) with the Bayesian R package *brms* (Bürkner, 2017). As most variables were ordered, instead of continuous, we did not centre or standardise the variables.

For all the models, we ran four chains of 5000 iterations each, which was enough to ensure convergence between the chains. We used the default priors in *brms* in all of the models. For all intercepts and all standard deviations, the prior was a Student *t* Distribution with 3 degrees of freedom and a standard deviation of 2.5 For all β coefficients, the prior was flat. For all the monotonic effects, the prior followed Dirichlet distribution with equal distance between all adjacent categories.

### 2.3. Results

#### 2.3.1. Which variables predict preference for a dominant leader?

To analyse the relationship between the predictors of interest and the preference for a dominant political leader, we ran several Bayesian regression models (Table 2) with default flat priors in *brms* and compared their model fit using leave-one-out cross validation information criterion (LOOIC; Vehtari et al., 2017). Similar to Akaike Information Criterion (AIC) and Watanabe–Akaike Information Criterion (WAIC), the absolute LOOIC value is not informative, but LOOIC values for similar models can be compared relative to one another to compare model fit to the data. A lower LOOIC indicates better model fit. First, we ran a null model (LOOIC = 132040.6, SE = 212.0), with only the intercepts as predictors. The variance ratio (a Bayesian equivalent to the intraclass correlation) in this null model was 0.06, meaning that 6% of the variance in preferences for dominant leaders is explained by the clustering of respondents within countries. This is a relatively small value but important enough to justify the use of multilevel modelling to attain accurate estimates. This model was compared with a control model (LOOIC = 131955.3, SE = 212.6), which included all of the control variables. As the model fit of the control model was better than the fit of the null model, we used the control model as a base for constructing and comparing the model fit of subsequent models.

**Table 2.** Unstandardised coefficients (*B*) and their standard errors (SE, in brackets) for each of the main ordinal regression models with preference for a dominant leader as the outcome. Square brackets indicate reference categories for the categorical predictors. Ordinal predictors were modelled as monotonic effects and are labelled ‘mo(variable)'. More regression models and further details can be found in the Supplementary Materials

Unstandardised coefficients	Null, *B* (SE)	Control, *B* (SE)	Economic uncertainty, *B* (SE)	Lack of control, *B* (SE)	Intergroup conflict, *B* (SE)	Full, *B* (SE)
Intercept [1]	−1.11 (0.12)	−1.00 (0.12)	−0.53 (0.13)	−0.94(0.13)	−0.68 (0.13)	−0.40 (0.13)
Intercept [2]	0.23 (0.12)	0.35 (0.12)	0.83 (0.13)	0.40 (0.13)	0.68 (0.13)	0.96 (0.13)
Intercept [3]	1.86 (0.12)	1.98 (0.12)	2.46 (0.13)	2.03 (0.13)	2.31 (0.13)	2.59 (0.13)
Gender [Male]		−0.03 (0.02)	−0.04 (0.02)	−0.03 (0.02)	−0.02 (0.02)	−0.03 (0.02)
Age		−0.00 (0.00)	0.00 (0.00)	−0.00 (0.00)	−0.00 (0.00)	−0.00 (0.00)
mo(Subjective Social Class)		0.12 (0.02)	0.13 (0.02)	0.12 (0.02)	0.12 (0.02)	0.13 (0.02)
mo(Income Category)		−0.00 (0.01)	0.01 (0.00)	−0.00 (0.01)	−0.00 (0.01)	0.01 (0.00)
mo(Political Ideology)		0.02 (0.00)	0.02 (0.00)	0.02 (0.00)	0.02 (0.01)	0.02 (0.00)
mo(EcUnJOB)			0.05 (0.01)			0.05 (0.01)
mo(EcUnEDUCATION)			0.04 (0.01)			−0.02 (0.01)
mo(EcUnFOOD)			0.15(0.02)			0.15 (0.02)
mo(EcUnMEDICINE)			0.06(0.01)			0.06 (0.01)
mo(EcUnCASH)			−0.04 (0.01)			−0.04 (0.01)
mo(Perceived Lack of Control)				0.01 (0.00)		0.00 (0.00)
mo(InConINTWAR)					0.07 (0.01)	0.06 (0.01)
mo(InConCIVILWAR)					0.12 (0.01)	0.11 (0.01)
mo(InConTERRORISM)					−0.06 (0.01)	−0.05 (0.01)
LOOIC	132,040.6	131,955.3	131,509.0	131,940.8	131,669.3	131,335.1
Variance ratio	0.12					

LOOIC, Leave-one-out cross validation information criterion (lower values indicate better fit to the data; see text for details). Variance ratio represents the proportion of variance explained by the clustering of individuals within states.

To test H1a, which predicted that economic uncertainty is positively related to preferences for a dominant leader, we ran a model that included the five items for economic uncertainty and the control variables (LOOIC = 131509.0, SE = 215.5). Supporting H1a, this economic uncertainty model had a better fit than the control model. Four items (EcUnJOB, *B* = 0.05, SE = 0.01, 89% CI [0.04, 0.06]; EcUnEDUCATION, *B* = 0.04, SE = 0.01, 89% CI [0.02, 0.06]; EcUnFOOD, *B* = 0.15, SE = 0.02, 89% CI [0.12, 0.18]; EcUnMEDICINE, *B* = 0.06, SE = 0.01, 89% CI [0.04, 0.07]) were, as expected, positively related to preference for a dominant leader, while one item (EcUnCASH, *B* = −0.05, SE = 0.01, 89% CI [−0.07, −0.04]) was, contrary to expectations, negatively related to preference for a dominant leader.

To test H2a, which predicted that perceived lack of control is positively related to preferences for a dominant leader, we ran a model that included perceived lack of control and the control variables (LOOIC = 131940.8, SE = 212.8). This model had a better fit than the control model. However, the fit of this model was worse than the fit of the economic uncertainty model, which indicates that perceived lack of control was less important in predicting preference for a dominant leader than economic uncertainty. The addition of perceived lack of control to the economic uncertainty model did not improve the latter's model fit (LOOIC = 131509.8, SE = 215.5) and the credible interval for perceived general lack of control crossed zero (*B* = 0.01, SE = 0.01, 89% CI [−0.01, 0.01]), indicating an unreliable effect of perceived lack of control on preferences for a dominant leader.

To test H3a, which predicted a positive relationship between intergroup conflict and preference for a dominant leader, we ran a model that included the three intergroup conflict items and the control variables (LOOIC = 131669.3, SE = 215.1). Supporting H3a, the intergroup conflict model had a better fit than the control model. Two of the intergroup conflict items (InConINTWAR, *B* = 0.07, SE−0.01, CI 89% [0.05, 0.09]; InConCIVILWAR, *B* = 0.12, SE = 0.01, 89% CI [0.10, 0.14]) were, as expected, positively related to preference for a dominant leader, while one item (InConTERRORISM, *B* = −0.06, SE = 0.01, 89% CI [−0.08, −0.04]) was, contrary to expectations, negatively related. However, the fit of these models was worse than the fit of the economic uncertainty model, which indicates that intergroup conflict had less importance than economic uncertainty in predicting preference for a dominant leader.

Lastly, we ran a full model including all the variables. This model had the best fit of all models (LOOIC = 131335.1, SE = 217.0). This indicates that, although economic uncertainty is a stronger predictor than intergroup conflict, intergroup conflict is still an important predictor of preference for a dominant leader. In the full model, three of the economic uncertainty variables were positively associated with preference for a dominant leader (EcUnJOB, *B* = 0.05, SE = 0.01, 89% CI [0.03, 0.06]; EcUnFOOD, *B* = 0.15, SE = 0.02, 89% CI [0.12, 0.17]; EcUnMEDICINE, *B* = 0.06, SE = 0.01, 89% CI [0.04, 0.08]), while two of the economic uncertainty variables were negatively related (EcUnEDUCATION, *B* = −0.02, SE = 0.01, 89% CI [−0.03, −0.01; EcUnCASH, *B* = −0.04, SE = 0.01, 89% CI [−0.06, −0.02]); two of the intergroup conflict variables were positively related to preference for a dominant leader (InConINTWAR, *B* = 0.06, SE = 0.01, 89% CI [0.04, 0.08]; InConCIVILWAR, *B* = 0.11, SE = 0.01, 89% CI [0.09, 0.13]), while one was negatively related (InConTERRORISM, *B* = −0.05, SE = 0.01, 89% CI [−0.07, −0.03]). Perceived lack of control had an unreliable effect on preference for a dominant leader as its credible interval crossed zero (*B* = 0.00, SE = 0.00, 89% CI [−0.00, 0.01]).

#### 2.3.2. Which variables predict preference for a prestigious leader?

Here we ran the same models as for preference for a dominant leader but with preference for a prestigious leader as outcome variable (Table 3). The null model (LOOIC = 131903.6, SE = 214.2) had a Variance Ratio of 0.06, justifying the use of multilevel modelling. Again, this null model had worse fit than the control model (LOOIC = 131641.5, SE = 215.7). Consequently, we used the control model as a base for constructing and comparing the model fit of the subsequent models.

**Table 3.** Unstandardised coefficients (*B*) and their standard errors (in brackets) for each of the main ordinal regression models with preference for a prestigious leader as the outcome. Square brackets indicate reference categories for the categorical predictors. Ordinal predictors were modelled as monotonic effects and are labelled mo(variable). More regression models and further details can be found in the Supplementary Materials

Unstandardised coefficients	Null B (SE)	Control B (SE)	Economic uncertainty B (SE)	Lack of control B (SE)	Intergroup conflict B (SE)	Full B (SE)
Intercept [1]	−1.92 (0.09)	−2.06 (0.09)	−1.65 (0.10)	−2.07 (0.09)	−1.80 (0.09)	−1.60 (0.10)
Intercept [2]	−0.37 (0.09)	−0.51 (0.09)	−0.09 (0.10)	−0.52 (0.09)	−0.25 (0.09)	−0.04 (0.10)
Intercept [3]	1.60 (0.09)	1.46 (0.09)	1.89 (0.10)	1.45 (0.09)	1.73 (0.09)	1.94 (0.10)
Gender [Male]		0.02 (0.02)	0.02 (0.02)	0.02 (0.02)	0.03 (0.02)	0.03 (0.02)
Age		−0.00 (0.00)	−0.00 (0.00)	−0.00 (0.00)	−0.00 (0.00)	−0.00 (0.00)
mo(Subjective Social Class)		−0.03 (0.04)	0.03 (0.04)	−0.04 (0.04)	−0.03 (0.04)	0.02 (0.04)
mo(Income Category)		0.22 (0.10)	0.29 (0.08)	0.21 (0.10)	0.26 (0.09)	0.30 (0.08)
mo(Political Ideology)		−0.11 (0.03)	−0.11 (0.03)	−0.11 (0.03)	−0.12 (0.03)	−0.12 (0.03)
mo(EcUnJOB)			0.05 (0.03)			0.02 (0.03)
mo(EcUnEDUCATION)			−0.03 (0.04)			−0.02 (0.04)
mo(EcUnFOOD)			0.29 (0.03)			0.22 (0.03)
mo(EcUnMEDICINE)			0.19 (0.04)			0.18 (0.04)
mo(EcUnCASH)			0.14 (0.04)			0.14 (0.04)
mo(Perceived Lack of Control)				−0.03 (0.04)		−0.01 (0.00)
mo(InConINTWAR)					0.16 (0.04)	0.10 (0.04)
mo(InConCIVILWAR)					0.05 (0.05)	−0.03 (0.05)
mo(InConTERRORISM)					0.15 (0.03)	0.15 (0.04)
LOOIC	131,903.6	131,641.5	131,600.2	131,864.0	131,704.8	131,541.0
Variance ratio	0.06					

Supporting H1b, the economic uncertainty model (LOOIC = 131600.2, SE = 216.2) had a better fit than the control model. Four items were, as expected, positively related to preference for a prestigious leader (EcUnJOB, *B* = 0.05, SE = 0.01, 89% CI [0.04, 0.07]; EcUnEDUCATION, *B* = 0.04, SE = 0.01, 89% CI [0.02, 0.06]; EcUNFOOD, *B* = 0.15, SE = 0.02, 89% CI [0.12, 0.18]; EcUnMEDICINE, *B* = 0.06, SE = 0.01, 89% CI [0.04, 0.08]), while one item (EcUnCASH, *B* = −0.04, SE = 0.01, 89% CI [−0.06, −0.02]) was, contrary to expectations, negatively related.

Contrary to H2b, the lack of control model (LOOIC = 131864, SE = 214.7) had worse fit than the control model. The inclusion of perceived lack of control in the economic uncertainty model hardly improved its model fit (LOOIC = 131600.2). Contrary to H3b, the intergroup conflict model (LOOIC = 131704.8, SE = 215.8) had worse fit than the control model.

The full model including all of the variables (LOOIC = 131541.0, SE = 216.6) had the best fit of all the models. In the full model, three of the economic uncertainty items were positively related to preference for a prestigious leader (EcUnJOB, *B* = 0.05, SE = 0.01, 89% CI [0.03, 0.06]; EcUnFOOD, *B* = 0.15, SE = 0.02, 89% CI [0.11, 0.18]; EcUnMEDICINE, *B* = 0.06, SE = 0.01, 89% CI [0.04, 0.08]), while one item was negatively related (EcUnCASH, *B* = −0.04, SE = 0.01, 89% CI [−0.06, −0.01]) and another item had an unreliable effect (EcUnEDUCATION, *B* = −0.02, SE = 0.01, 89% CI [−0.04, 0.01]); two of the intergroup conflict items were positively related to preference for a prestigious leader (InConINTWAR, *B* = 0.06, SE = 0.01, 89% CI [0.03, 0.08]; InConCIVILWAR, *B* = 0.11, SE = 0.01, 89% CI [0.08, 0.13]), while one item was negatively related (InConTERRORISM, *B* = −0.05, SE = 0.01, 89% CI [−0.08, −0.03]). Perceived lack of control had an unreliable effect of preference for a prestigious leader (*B* = −0.01, SE = 0.00, 89% CI [−0.01, 0.00]).

### 2.4. Discussion

In Study 1, we tested whether economic uncertainty, perceived lack of control, and intergroup conflict positively predict preference for dominant and prestigious leaders. Previous research has focused on how these variables predict preference for a dominant leader but, to the best of our knowledge, no research has tested how these variables predict preference for a prestigious leader. Moreover, previous studies with data from the WVS (Kakkar & Sivanathan 2017) used group-level measures of the predictor variables, whereas we used individual-level measures, providing a more fine-grained analysis.

Similarly to Kakkar and Sivanathan (2017), we found that some of our measures of economic uncertainty predicted preference for a dominant leader. In our study, however, the same measures of economic uncertainty also predicted preference for a prestigious leader. The fact that economic uncertainty is related to preferences for both types of leaders casts doubt on previous claims of a specific link between economic uncertainty and preference for dominant leaders. Our results suggest that economic uncertainty might simply increase preference for leadership in general, instead of for dominant leadership in particular.

Alternatively, the relationship between economic uncertainty and preference for both dominant and prestigious leaders might be mediated or moderated by respondents’ traits or states. Here, we examined the relationship between one of these individual variables, perceived general lack of control, and preference for dominant and prestigious leaders. Perceived general lack of control has been proposed to be positively related to preference for a dominant leader and to be the psychological mechanism by which facing economic uncertainty makes an individual more likely to prefer a dominant leader (Kakkar & Sivanathan, 2017). We therefore proposed that perceived lack of control would also be positively related to preference for prestigious leaders. However, we did not find support for any of these predictions as perceived lack of control had an unreliable effect on predicting preference for both dominant and prestigious leaders. Consequently, perceived lack of control does not seem to be the mechanism that explains the higher preference for both dominant and prestigious leaders when facing economic uncertainty.

Similarly to previous studies (Laustsen & Petersen, 2017; Little et al., 2007), we found that some of our measures of intergroup conflict predicted preference for a dominant leader. Although the fit of the intergroup conflict model for predicting preference for a prestigious leader was worse than the fit of the control model, two intergroup conflict items were positively related to preference for a prestigious leader in the full model, which had the best fit overall. This again casts doubt on the specificity of the relationship between intergroup conflict and preference for a dominant leader. As we suggest above, dominant behaviours directed against outgroups might serve to gain prestige within the ingroup (Halevy et al., 2012). This explains why people might prefer an authoritarian over a democratic government when political repression is exercised against outgroups considered enemies of the ingroup (e.g. political dissidents in Stalin's USSR). Although further research is necessary to confirm this, when respondents are asked about their preferences for dominant and prestigious leaders, it is likely that respondents are imagining that the political authoritarianism and the expertise would be used in favour of the ingroup and/or against outgroups.

Compared with previous studies, our study has the advantage of comparing preferences for different types of leadership (dominance vs prestige) when studying the effects of specific social contexts such as economic uncertainty or intergroup conflict on preferences for one type of leader. Another advantage is the simultaneous use of different measures of economic uncertainty and intergroup conflict. As the results suggest, not all measures of these variables are positively related to preferences for dominant and prestigious leaders. It seems that being worried about not having or finding a job, and not having had enough food and medicine in the last 12 months are stronger predictors on preferences for dominant and prestigious leaders than being worried about access to education and not having enough income. Similarly, open intergroup conflict (inter-country or civil war) positively predict preference for dominant and prestigious leaders, while more unidirectional violence (terrorism) is negatively related to preference for both types of leaders. We are not sure why these different economic uncertainty and intergroup conflict variables are related to preferences for both types of leaders in different directions. However, the results make clear that selecting only some of these variables might bias the conclusions of studies investigating the relationship between particular economic and intergroup contexts and preferences for different types of leaders. Consequently, we recommend using multiple proxies for economic uncertainty and intergroup conflict in future studies.

Study 1 has the limitation of using measures of dominant leaders (‘strong leader who does not bother with parliament or elections’) and prestigious leaders (‘experts, not government, [who] make decisions according to what they think is best for the country’) that describe dictators and technocrats respectively. Dictatorship and technocracy are not incompatible forms of ruling a country. For example, technocrats occupied ministries and had special relevance in Franco's dictatorship in the 1960s in Spain. Moreover, these measures (dictator/technocrat) are not totally comparable with the measures of dominance and prestige commonly used to study the dual evolutionary model of social hierarchy such as the scale developed by Cheng et al. (2010). We addressed this problem in Study 2.

## 3. Study 2

### 3.1. Introduction

In Study 2, we first analyse how political ideology influences perceptions of political leaders as dominant or prestigious (H4 and H5; see Table 1). Following Kakkar and Sivanathan (2017), we use self-ratings of political ideology and ratings of the perceived dominance and prestige of Donald Trump and Hillary Clinton collected during the campaigns for the 2016 US Presidential Elections. American politics provides a particularly clear ingroup vs outgroup within-country comparison, with only two major political parties (Democrats and Republicans) represented by single candidates (in 2016, Clinton and Trump respectively) that are divided on many political and social issues. In line with H4, we expect to find that liberal ideology is positively related to perceptions of Trump as dominant and negatively related to perceptions of Clinton as dominant. In line with H5, we expect to find that liberal ideology is positively related to perceptions of Clinton as prestigious and negatively related to perceptions of Trump as prestigious. Second, we compare the strength of political ideology and economic uncertainty in predicting preferences for Donald Trump or Hillary Clinton. Following H6, we expect to find that political ideology is a stronger predictor of voting decision than economic uncertainty.

While H4 and H5 are unexplored in previous research, H6 has been explicitly addressed by Kakkar and Sivanathan (2017). In a pretest to their Study 1, they asked 120 Amazon Mechanical Turk participants to rate the prestige (agreement with statements such as ‘I think compared to Hillary Clinton, Donald Trump is a kind of leader who is respected and admired by other members’) and dominance (agreement with statements such as ‘I think compared to Donald Trump, Hillary Clinton is a kind of leader who might be feared by some members’) of both candidates using an adapted shorter version of a validated scale of prestige and dominance (Cheng et al., 2010). Agreement was rated on a Likert scale from 1 (not at all) to 7 (very much). Participants also rated their own political ideology on a seven-point Likert scale from 1 (conservative/Republican) to 7 (liberal/Democrat), but the authors did not use this for their analysis. The data was collected during the day of the third presidential debate on 20 October 2016. They found that the ratings of dominance were significantly higher for Trump (*M* = 5.5, SD = 1.5) than for Clinton (*M* = 4.7, SD = 1.8), while the ratings of prestige were higher for Clinton (*M* = 4.7, SD = 1.7) than for Trump (*M* = 3.54, SD = 1.87), leading to Kakkar and Sivanathan equating Donald Trump with a dominant leader.

On the same day, they asked 750 Amazon Mechanical Turk participants about their intention to vote for Donald Trump, Hillary Clinton or neither, as well as their political ideology using the scale described above. They measured economic uncertainty using an aggregated measure of the rates of unemployment, house vacancy and poverty within the ZIP code of each participant, extracted from the Distress Community Index (Economic Innovation Group, 2016). A multinomial regression with economic uncertainty, political ideology, and several control variables showed that economic uncertainty was positively related to preference for Trump over Clinton. As the coefficient of economic uncertainty was larger than the coefficient for political ideology they concluded that ‘economic uncertainty predicted a preference for Donald Trump over and above (…) political partisanship’ (p. 6736). However, their coefficients were not standardised and, therefore, their conclusion might be misleading. In fact, the difference in proportion of variance explained by their models with economic uncertainty (adjusted *R*^2^ = 0.227) and without economic uncertainty (adjusted *R*^2^ = 0.222) is only 0.5%, which diminishes the importance of economic uncertainty in predicting voting intention for Trump. In our Study 2, we use the data from Kakkar and Sivanathan. Like those authors, we conducted multinomial regressions. We adopted a model comparison approach to make more reliable comparisons between the strength of economic uncertainty and political ideology, rather than comparing unstandardised coefficients. As we did not find a way to run multinomial Bayesian regression using ordered categorical predictors, we ran frequentist models using AIC instead of LOOIC for the model comparisons and treated ordered categorical variables as if they were continuous. A difference of at least 2 AICs is considered to constitute a reliable difference between models in their fit to the data. We assume that models with AICs that differ by less than 2 do not differ in their ability to explain the data. All of the models were run in Stata 16 (Stata Corp, 2019).

### 3.2. Methods

For testing H4 and H5, we used the data from the sample of 120 participants in Kakkar and Sivanathan (2017). For testing H6, we used the data from the sample of 750 participants in Kakkar and Sivanathan (2017). We also tested H6 using the actual results of the US Presidential Elections of 2016. To this end, we conducted binomial regressions in which Donald Trump's victory within each state was predicted by the level of economic uncertainty within the state and the percentage of votes obtained by the Republicans in previous Presidential Elections (2012) as a proxy for political ideology.

### 3.3. Results

**Figure 1.**
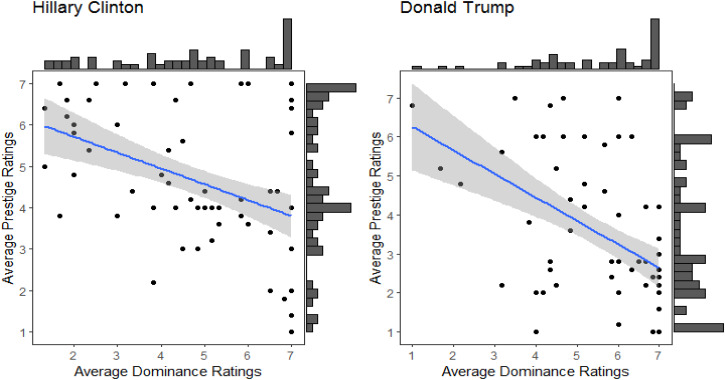
Relationship between the average dominance ratings and average prestige ratings for each candidate with 89% confidence intervals (grey area) and marginal histograms. Left: average dominance ratings and average prestige ratings for Hillary Clinton. Right: average dominance ratings and average prestige ratings for Donald Trump.

As prestige and dominance have been conceptualised as two separate constructs (see Section 1.2) and the results of previous studies have shown that prestige and dominance barely correlate (Brand & Mesoudi, 2019; Cheng et al., 2010, 2013; Kakkar et al., 2020; Monge-López & Álvarez-Solas, 2017; Redhead et al., 2018a), we first explored whether averaged ratings of prestige and dominance for each political candidate were correlated. Contrary to previous studies, we found a moderate negative correlation between the ratings of dominance and prestige for both candidates (Clinton, *r* = −0.42; Trump, *r* = −0.48; see Figure 1).

**Figure 2.**
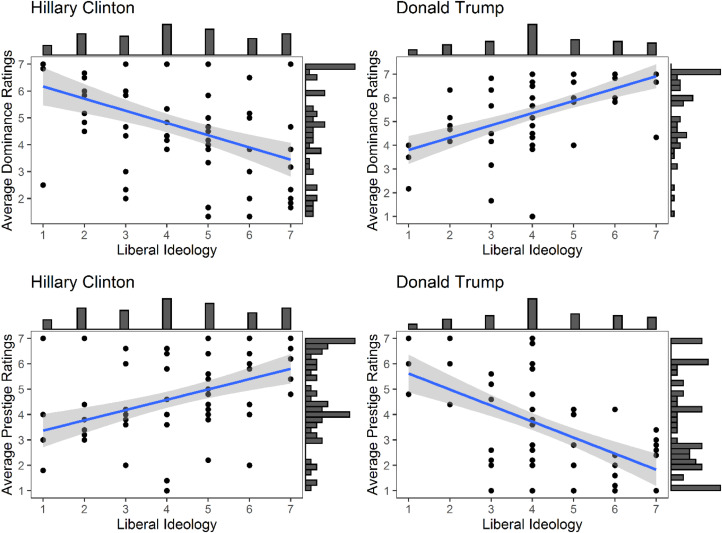
Relationship between the average prestige ratings and average dominance ratings for each candidate and political ideology of participants on a scale from 1 (conservative/Republican) to 7 (liberal/Democrat) with 89% *confidence intervals* (grey areas) and marginal histograms. Left, Clinton; *right*, Trump.

Supporting H4, we found that liberal ideology was positively related to ratings of Trump as dominant (*r* = 0.57) and negatively related to ratings of Clinton as dominant (*r* = −0.45). Supporting H5, we found that liberal ideology was positively related to ratings of Clinton as prestigious (*r* = 0.44) and negatively related to ratings of Trump as prestigious (*r* = −0.56). See Figure 2.

To test H6, that political ideology is a stronger predictor of voting decisions than economic uncertainty, we carried out a number of multinomial logistic regression models (Finch et al., 2014, pp. 131–133) with voting for neither Trump nor Clinton as the reference category (Table 4). First, we compared the fit of a null model with and without intercepts varying by state. The fit of the single-level null model (AIC = 1541.978) was better than the fit of the multilevel null model (AIC = 1544.296). Consequently, multilevel modelling was not necessary here. All of the subsequent models are single-level models.

**Table 4.** Multinomial regressions with neither Trump nor Clinton as reference category. Standard Errors are shown in parentheses. The model comparisons using the AIC show that the political ideology model (in bold) has the best fit to the data

Unstandardised coefficients	Null	Control	Economic uncertainty	Political ideology	Full
*Vote for Clinton*					
Constant	0.67 (0.09)	−0.47 (0.41)	0.68 (0.63)	−3.57 (0.57)	−2.47 (0.20)
Gender [Female]		0.45 (0.18)	0.44 (0.18)	0.27 (0.20)	0.25 (0.20)
Age		0.01(0.00)	0.00 (0.01)	0.01 (0.01)	0.01(0.01)
Income		0.14 (0.06)	0.11 (0.06)	0.17 (0.07)	0.14 (0.07)
Poverty			−1.61 (1.21)		−2.42 (1.31)
Unemployment			−2.21 (1.35)		−1.06 (1.44)
Housing vacancy			1.41 (2.68)		0.53 (2.90)
Liberal ideology				0.62 (0.07)	0.62(0.07)

*Vote for Trump*					
Constant	−0.21 (0.10)	−1.51 (0.49)	−1.41 (0.76)	1.07 (0.62)	1.489 (0.90)
Gender [Female]		−0.24 (0.23)	−0.23 (0.23)	−0.21 (0.25)	−0.16 (0.25)
Age		0.04 (0.01)	0.03 (0.01)	0.03 (0.01)	0.03 (0.01)
Income		0.13 (0.07)	0.13 (0.07)	0.08 (0.08)	0.07 (0.08)
Poverty			−1.77 (1.47)		−0.96 (1.57)
Unemployment			−0.58 (1.61)		−1.91 (1.78)
Housing vacancy			5.87 (3.08)		7.29 (3.39)
Liberal ideology				−0.59 (0.08)	−0.60 (0.08)
AIC	1541.98	1518.95	1516.06	1223.04	1220.08
Pseudo-*R*^2^		0.03	0.03	0.22	0.23

Second, we compared the fit of the null model with a control model, which included age, gender and income as predictors (AIC = 1518.949). Given its better fit, we used the control model as a base for the following models. Third, we compared the control model with a model that also included separately the three economic uncertainty variables poverty rate, unemployment rate and housing vacancy rate. The model fit of this economic uncertainty model (AIC = 1516.061) was slightly better than the control model. However, a model with the control variables and political ideology as predictors had considerably better fit (AIC = 1283.126) than the economic uncertainty model.

Lastly, a full model was computed, which had the best fit of all models (AIC = 1220.082). These results therefore support H6, i.e. political ideology is a stronger predictor of voting decision than economic uncertainty. In the full model, liberal ideology positively predicted preference for Clinton (*B* = 0.62, SE = 0.07, 89% CI [0.51, 0.73]) and negatively predicted preference for Trump (*B* = −0.60, SE = 0.08, 89% CI [−0.73, −0.47). Among the economic uncertainty variables, only one, housing vacancy rate, supports a greater preference for Trump (*B* = 7.29, SE = 3.39, 89% CI [1.87, 12.70]) than for Clinton (*B* = 0.52, SE = 2.90, CI [−4.10, 5.16]) when facing economic uncertainty. Poverty rate is negatively related to both preferences for Clinton (*B* = −2.42, SE = 1.31, CI 89% [−4.52, −0.32]) and Trump (*B* = −0.96, SE = 1.57, 89% CI [−3.47, 1.55]). However, the CI for Trump crosses zero, indicating that the negative relationship is not reliable. Unemployment rate is also negatively related to preferences for both Clinton (*B* = −1.06, SE = 1.44, 89% CI [−3.37, 1.25]) and Trump (*B* = −1.90, SE = 1.78, 89% CI [−4.76, 0.94]). For both candidates, this negative relationship is not reliable as both CIs cross zero.

Four alternative statistical procedures were conducted to confirm these results (see Supplementary Material). In all of these, we found that political ideology was a stronger predictor of voting decision than economic uncertainty.

Finally, we tested H6 by comparing how well economic uncertainty and political ideology predicted the actual victory of Donald Trump in the 2016 Presidential Election in each State (Table 5). We compared a null model (AIC = 69.30) with a model with the economic uncertainty variables at the level of the State (AIC = 67.30), a model with political ideology measured as the percentage of votes for Republicans in the elections of 2012 (AIC = 19.37) and a full model that included both economic uncertainty and political ideology (AIC = 20.52). Although the economic uncertainty model improved the fit of the null model, both the political ideology model and the full model had a better fit to the data. In the political ideology model, the percentage of votes for Republicans in 2012 positively predicted the victory in 2016 of Donald Trump in a State (*b* = 0.81, SE = 0.38, 89% CI [0.19, 1.44]). Three alternative statistical procedures were conducted to confirm these results (see Supplementary Material). In all of these, we found that political ideology was a stronger predictor of voting decision than economic uncertainty.

**Table 5.** Binomial regressions predicting the victory of Donald Trump within each State of the US in the 2016 Presidential Elections. The model comparisons using the AIC show that the political ideology model (in bold) has the best fit to the data

Unstandardised coefficients	Null	Economic uncertainty	Political ideology	Full
Constant	0.41 (0.29)	4.70 (4.53)	−37.61 (17.63)	−149.89 (96.21)
Housing		0.77 (0.30)		−1.55 (1.23)
Unemployment		−0.45 (0.17)		0.15 (0.42)
Poverty		0.55 (0.25)		1.62 (1.25)
Political ideology			0.81 (0.38)	2.89 (1.88)
AIC	69.30	67.30	19.37	20.52
Pseudo-*R*^2^		0.35	0.77	0.84

### 3.4. Discussion

In Study 2, we first examined how political ideology influences ratings of prestige and dominance of political leaders, using Donald Trump and Hillary Clinton as stimuli. Second, we compared the strength of political orientation and economic uncertainty in predicting preference for Trump or Clinton.

Contrary to previous studies which found that ratings of dominance and prestige for the same individuals are uncorrelated, the ratings of dominance and prestige for both Trump and Clinton were negatively correlated. That is, the higher a respondent rated Trump as dominant, the lower they rated him as prestigious, and vice versa. Similarly, the higher a respondent rated Clinton as dominant, the lower they rated her as prestigious, and vice versa. This might be due to the polarised attitudes towards both candidates in the US at the time of collecting the data. Supporting this, we found that ratings of both dominance and prestige were influenced by political ideology. As predicted by H4 and H5, liberal ideology was positively correlated with ratings of Trump as dominant and Clinton as prestigious, and negatively correlated with ratings of Hillary as dominant and Trump as prestigious.

This highlights the importance of studying the relationship between individual variables such as political ideology with dominance and prestige before concluding that a politician is either dominant or prestigious. Kakkar and Sivanathan (2017) concluded that Trump was a dominant leader and Clinton a prestigious leader because ANOVAs comparing the ratings of dominance and prestige of both candidates yielded *p*-values smaller than 0.05. However, as the variation in the ratings was related to political ideology, the inferences about the preferences for dominant or prestigious political leaders in this context is misleading. Consequently, future studies should pay careful attention to potential systematic variation in the perceptions of dominance and prestige of the stimuli to avoid potential misleading inferences (see Mileva et al., 2016, p. for another example of variation of perceptions of dominance and prestige related to participants' characteristics). This is especially important when studying political issues, as the lack of political diversity in social science disciplines such as psychology, which is heavily skewed towards the left (Langbert et al., 2016), might lead to less questioning of research methods that yield results in agreement with researchers’ own political views (Clark & Winegard, 2020; Duarte et al., 2015; Martin, 2016), as well as biased results if using samples that are more left-leaning than the general population, such as Amazon Mechanical Turk participants (Clifford et al., 2015) or psychology undergraduates.

Although the results of Study 2 show that perceptions of dominance and prestige of both candidates are associated with participants’ political ideologies, this does not mean that Clinton and Trump did not differ in their use of the dominance and prestige strategies during the presidential debates. Indeed, Witkower (2017) found that Clinton showed more demonstrations of knowledge and exhibited more prestige-related non-verbal displays such as smiling than did Trump, while Trump made more verbal attacks and showed more dominance-related non-verbal displays such as occupying more space and extending his arms than did Clinton. As argued earlier, the use of the dominance strategy against outgroups (which for Republicans would be Democrats) might lead to higher prestige among members of the ingroup. Experimental evidence, however, is necessary to test this prediction.

Supporting H6, our results also contradict Kakkar and Sivanathan's conclusion that economic uncertainty is a stronger predictor of voting for Trump than political ideology. This is because we conducted model comparisons taking in and out both predictors, which we considered more appropriate for comparing the relative strength of economic uncertainty and political ideology than comparing unstandardised coefficients. Political ideology was also a stronger predictor than economic uncertainty in predicting preference for Donald Trump when we used the data for the actual 2016 US Elections.

A limitation of our study, however, is that the data does not include education and race as predictors of voting preference, which are factors that seem to have played a role in determining the outcome of the 2016 US Presidential Election (Mutz, 2018b). Another limitation is that the study did not include any measure of whether the popularity of Donald Trump in the 2016 elections was motivated by a cultural backlash against post-materialist values and political correctness, as participants were not asked about these issues. Furthermore, when people decide to vote for a specific candidate in the elections it is also possible that they move towards the candidate's ideology, which would explain why political ideology is such a strong predictor of voting decision. Supporting this, the results of a longitudinal study show that people tended to vote for the candidates of the same party in the US Presidential Elections of 2012 and 2016 but that from one to the other there was a slight but important change in party identification in favour of the Republican party (Mutz, 2018b). The same study also found that personal economic hardship including subjective judgement of the economic situation did not predict voting for Trump. However, increases in Social Dominance Orientation, which is related to preference for group-based dominance, positively predicted voting for Trump. The results of Mutz's study are congruent with Inglehart and Norris’ cultural backlash hypothesis and, as in our study, they diminish the importance of economic uncertainty in predicting preference for Trump over Clinton (but see debate about the correct way to analyse and interpret the data; Morgan, 2018a, b; Mutz, 2018a).

## 4. General discussion

In this article, we first reviewed how the dual evolutionary model of social hierarchy has been used to explain the rise in popularity and electoral victories of right-wing populist leaders such as Donald Trump. Second, we highlighted the limitations of applying this model to large-scale democratic societies without clearly distinguishing between ingroups and outgroups. Third, we showed that economic uncertainty and intergroup conflict predict preference for *both* dominant and prestigious leaders using data from the WVS. Fourth, we showed that perceptions of political leaders as either dominant or prestigious are not universal, but depend on people's political ideologies. Conservatives perceive conservative political leaders as prestigious and liberal political leaders as dominant, while liberals perceive conservative political leaders as dominant and liberal political leaders as prestigious. This highlights the importance of distinguishing between ingroups and outgroups within societies when reaching conclusions about preferences for dominant or prestigious leaders. Fifth, we showed that political ideology is a stronger predictor of preference for Donald Trump and Hillary Clinton than economic uncertainty, contradicting previous conclusions attributing greater importance to the economy than ideology in explaining Donald Trump's victory in 2016 (see Kakkar & Sivanathan, 2017).

The main finding of Study 1 is that both economic uncertainty and intergroup conflict positively predict preference for both dominant and prestigious leaders. This result suggests that neither economic uncertainty nor intergroup conflict has a unique link with increased preferences for dominant leaders, as previous research has suggested (Kakkar & Sivanathan, 2017; Laustsen & Petersen, 2017; Little et al., 2007). Because one way to overcome hardship is through greater coordination and top-down decision-making, people might increase their preference for leadership in general, regardless of the type of leadership, under conditions of economic uncertainty and intergroup conflict.

Previous work has equated right-wing populist leaders with dominant leaders (e.g. Trump) and well-established liberal politicians (e.g. Clinton) with prestigious leaders (Kakkar & Sivanathan, 2017). However, Study 2 clearly shows that people perceive the dominance and prestige of political leaders differently depending on their own ideological similarity to those political leaders. Furthermore, as economic uncertainty does not seem to affect people's voting decision directly, we suggest looking at the political discourse of right-wing populist leaders and how it interacts with the discourse of other political actors, to explain their rise in electoral popularity (for further discussion see Jiménez, 2020, pp. 255–260). Previous research has shown that particular features of information such as being simple (Heath & Heath, 2008), concrete (Heath & Heath, 2008), emotional (Eriksson & Coultas, 2014; Heath et al., 2001; Heath & Heath, 2008; Stubbersfield et al., 2017) or negative (Bebbington et al., 2017) increases its chances of being transmitted accurately. As the right-wing populist discourse seems to contain these features in a greater proportion (e.g. higher emotional content; Breeze, 2018; Wirz, 2018) than the political discourse of traditional politicians, we suggest that this might explain the rise in electoral popularity of right-wing populist leaders. Nevertheless, the political discourse of part of the left (e.g. the emotional discourse of Greta Thunberg at the UN; PBS NewsHour, 2019) and against right-wing populist leaders (e.g. the emotional reaction after the election of Donald Trump as President of the US; Campbell & Manning, 2018, pp. viii–xix) sometimes presents the same content characteristics and is similarly simple, concrete, emotional, and negative. Consequently, we suggest that the study of the transmissibility of right-wing populist discourse and the discourse against right-wing populism should always take into account the political ideology of participants and the interaction between ideological groups.

In conclusion, while there have been prominent claims linking the rise of right-wing populist leaders, via economic uncertainty, to the dominance strategy of social rank acquisition and leadership, in this paper we have highlighted several limitations of these claims, alongside re-analyses and novel analyses to support our arguments. We hope to have contributed to continuing interdisciplinary efforts to improve our understanding of these major social and political trends that increasingly characterise our current times.

## Supplementary material

For supplementary material accompanying this paper visit https://doi.org/10.1017/ehs.2021.12. click here to view supplementary material

## Research transparency and reproducibility

Datasets and data analyses are available from https://github.com/AngelVJimenez/Preferences_Dominant_Prestigious_Leaders
