# When do people prefer dominant over prestigious political leaders? – ERRATUM

**DOI:** 10.1017/ehs.2021.24

**Published:** 2021-03-29

**Authors:** Ángel V. Jiménez, Adam Flitton, Alex Mesoudi

The Publisher apologises that upon publication of this paper the wrong graphical abstract was included.

The online version of this article has been updated with the correct image
