# Supplementary material for: When do people prefer dominant over prestigious political leaders?
Source: Evol Hum Sci. 2021 Mar 1;3:e16. doi: 10.1017/ehs.2021.12 (PMC10427292; doi:10.1017/ehs.2021.12)
Supplement: Supplementary file 1 [file S2513843X21000128sup001.pdf]

SUPPLEMENTARY MATERIALS

Alternative Analyses to predict preferences for Donald Trump and Hillary Clinton using the data by Kakkar and Sivanathan (2017)

Vote for Clinton					
**Unstandardized Coefficients**	**NULL**	**CONTROL**	**ECONOMIC UNCERTAINTY**	**POLITICAL IDEOLOGY**	**FULL**
**Constant**	0.67 (0.09)	-0.47 (0.41)	0.50 (0.53)	**-3.57 (0.57)**	-2.55 (0.67)
**Gender [Female]**		0.45 (0.18)	0.43 (0.18)	**0.27 (0.20)**	0.26 (0.20)
**Age**		0.01(0.00)	0.00 (0.01)	**0.01 (0.01)**	0.01 (0.01)
**Income**		0.14 (0.06)	0.12 (0.15)	**0.17 (0.07)**	0.14 (0.07)
**Economic Uncertainty**			-4.15 (1.45)		-4.37 (1.58)
**Political Ideology**				**0.62 (0.07)**	0.62 (0.07)
**Vote for Trump**					
**Unstandardized Coefficients**	**NULL**	**CONTROL**	**ECONOMIC UNCERTAINTY**	**POLITICAL IDEOLOGY**	**FULL**
**Constant**	-0.21 (0.10)	-1.51 (0.49)	-1.43 (0.64)	**1.07 (0.62)**	1.04 (0.76)
**Gender [Female]**		-0.24 (0.23)	-0.24 (0.22)	**-0.21 (0.25)**	-0.21 (0.25)
**Age**		0.04 (0.01)	0.04 (0.01)	**0.03 (0.01)**	0.03 (0.01)
**Income**		0.13 (0.07)	0.13 (0.07)	**0.08 (0.08)**	0.08 (0.08)
**Economic Uncertainty**			-0.33 (1.69)		-0.13 (1.79)
**Political Ideology**				**-0.59 (0.08)**	0.59 (0.08))
**AIC**	1541.98	1518.95	1512.40	**1223.04**	1217.88
**Pseudo-R^2**		0.03	0.03	**0.22**	0.22

Table 6. Alternative Analysis 1 to Predict Preferences for Donald Trump and Hillary Clinton. Economic uncertainty = (housing vacancy rate + unemployment rate + poverty rate) /3 measured at the zip code level. Multinomial model with fixed intercepts.

	NULL	CONTROL	ECONOMIC UNCERTAINTY	POLITICAL IDEOLOGY	FULL
**Vote for Trump**					
**Constant**	0.66 (0.09)	-0.47 (0.41)	-0.51 (0.53)	-3.57 (0.57)	**-2.53 (0.70)**
**Gender [Female]**		0.45 (0.18)	0.43 (0.18)	0.27 (0.20)	**0.26 (0.20)**
**Age**		0.00 (0.01)	0.00 (0.06)	0.01 (0.01)	**0.14 (0.07)**
**Income**		0.14 (0.06)	0.11 (0.06)	0.17 (0.07)	**0.62 (0.07)**
**Economic Uncertainty**			-4.17 (1.45)		**-4.41 (1.59)**
**Political Ideology**				0.61 (0.7)	
	**NULL**	**CONTROL**	**ECONOMIC UNCERTAINTY**	**POLITICAL IDEOLOGY**	**FULL**
**Vote for Clinton**					
**Constant**	-0.22 (0.12)	-1.51 (0.50)	-1.43 (0.65)	1.24 (0.66)	**1.24 (0.80)**
**Gender [Female]**		-0.25 (0.23)	-0.25 (0.23)	-0.21 (0.25)	**-0.20 (0.25)**
**Age**		0.04 (0.01)	0.03 (0.01)	0.03 (0.01)	**0.02 (0.01)**
**Income**		0.13 (0.07)	0.13 (0.07)	0.07 (0.08)	**0.07 (0.08)**
**Economic Uncertainty**			-0.32 (1.72)		-0.05 (1.89)
**Political Ideology**				-0.63 (0.09)	**-0.63 (0.09)**
**AIC**	1544.30	1521.69	1519.02	1221.42	**1220.79**

Table 7. Alternative Analysis 2 to Predict Preferences for Donald Trump and Hillary Clinton. Economic uncertainty = (housing vacancy rate + unemployment rate + poverty rate) /3 measured at the zip code level. Multinomial model with State as random intercept.

Vote for Clinton		Vote for Trump
Standardized Coefficients		
Constant	0.9 (0.30)	-0.54 (0.37)
Gender [Female]	0.28 (0.20)	-0.22 (0.25)
Age	0.06 (0.10)	0.26 (0.12)
Income	0.23 (0.10)	0.14 (0.12)
Duration Living in Zip Code	0.14 (0.10)	0.10 (0.12)
Total Population	0.05 (0.10)	-0.17 (0.12)
Density	-0.06 (0.11)	-0.08 (0.14)
Number of Zips in the County	0.09 (0.11)	0.11 (0.14)
Economic Uncertainty	-0.26 (0.10)	0.01 (0.11)
Political Ideology	1.08 (0.12)	-1.01 (0.14)
Pseudo-R^2	0.23	

Table 8. Alternative Analysis 3 to Predict Preference for Donald Trump and Hillary Clinton. Full model with standardized coefficients with same controls as Kakkar and Sivanathan (2017). Multinomial model with fixed intercept.

Vote for Clinton		Vote for Trump
Standardized Coefficients		
Constant	0.09 (0.30)	-0.62 (0.40)
Gender [Female]	0.28 (0.20)	-0.20 (0.25)
Age	0.06 (0.11)	0.27 (0.12)
Income	0.23 (0.10)	0.13 (0.13)
Duration Living in Zip Code	0.14 (0.10)	0.09 (0.12)
Total Population	0.05 (0.10)	-0.18 (0.13)
Density	-0.06 (0.11)	-0.10 (0.14)
Number of Zips in the County	0.09 (0.11)	0.22 (0.16)
Economic Uncertainty	-0.26 (0.10)	0.01 (0.11)
Political Ideology	1.08 (0.12)	-1.09 (015)

Table 9. Alternative Analysis 4 to Predict Preference for Donald Trump and Hillary Clinton. Full model with standardized coefficients with same controls as Kakkar and Sivanathan (2017). Multinomial model with random intercepts.

Alternative Analyses for Predicting Preference for Trump with the Data of the Actual Results of the 2016 US Presidential Elections

Unstandardized Coefficients	NULL	ECONOMIC UNCERTAINTY	POLITICAL IDEOLOGY	FULL
**Constant**	0.41 (0.29)	-6.29 (2.62)	**-37.61 (17.63)**	-49.57 (24.61)
**Economic Uncertainty**		0.31 (0.12)		0.32 (0.27)
**Political Ideology**			**0.81 (0.38)**	0.91 (0.48)
**AIC**	69.30	63.60	**19.37**	19.79
**Pseudo-R^2**		0.11	**0.77**	0.79

Table 10. Alternative Analysis 1 to Predict Trump's Victory within States. Logistic regression (outcome: 1=Trump's victory in a State, 0=Clinton's victory in a State. Proxy for political ideology = Percentage votes for Republicans in a State in 2012. Economic uncertainty variables (housing vacancy rate, unemployment rate and poverty rate) introduced as composite measure.

Unstandardized Coefficients	NULL	ECONOMIC UNCERTAINTY	POLITICAL IDEOLOGY	FULL
**Constant**	- 0.03 (0.28)	-1.13 (2.20)	**-1.85 (1.48)**	-2.16 (2.40)
**Economic Uncertainty**		0.05 (0.10)		0.02 (0.11)
**Political Ideology**			**0.04 (0.03)**	0.03 (0.03)
**AIC**	71.19	71.31	**61.52**	63.30
**Pseudo-R^2**		0.12	**0.79**	0.80

Table 11. Alternative Analysis 2 to Predict Trump's Victory within States. Logistic Regression for Proportional Data (outcome: percentage of votes for Trump in 2016). Proxy for political ideology = Percentage votes for Republicans in a State in 2012. Economic uncertainty = (housing vacancy rate + unemployment rate + poverty rate)/3.

Unstandardized Coefficients	NULL	HOUSING	UNEMPLOYMENT	POVERTY	ECONOMIC UNCERTAINTY	POLITICAL IDEOLOGY	FULL
**Constant**	- 0.03 (0.28)	0.94 (1.23)	-0.68 (2.93)	-0.81 (1.39)	0.84 (3.45)	**-1.85 (1.48)**	-1.58 (4.40)
**Housing**		0.11 (0.14)			0.14 (0.22)		0.03 (0.03)
**Unemployment**			0.01 (0.07)		-0.07 (0.12)		-0.07 (0.24)
**Poverty**				0.05 (0.09)	0.06 (0.17)		-0.01 (0.14)
**Political Ideology**						**0.04 (0.03)**	-0.00 (0.18)
**AIC**	71.19	68.20	72.98	70.504	68.56	**61.52**	66.12
**Pseudo-R^2**		0.28	0.02	0.16	0.44	**0.79**	0.84

Table 12. Alternative Analysis 3 to Predict Trump's Victory within States. Logistic Regression for Proportional Data (outcome: percentage of votes for Trump in 2016). Proxy for political ideology = Percentage votes for Republicans in a State in 2012. Economic uncertainty variables (housing vacancy rate, unemployment rate and poverty rate) introduced as separate predictors.

ADDITIONAL SUPLEMENTARY MATERIALS

All the data and R code can be found here:

https://github.com/AngelVJimenez/Preferences_Dominant_Prestigious_Leaders
